# The association between imitation recognition and socio-communicative competencies in chimpanzees (*Pan troglodytes*)

**DOI:** 10.3389/fpsyg.2015.00188

**Published:** 2015-02-26

**Authors:** Sarah M. Pope, Jamie L. Russell, William D. Hopkins

**Affiliations:** ^1^Neuroscience Institute and Language Research Center, Georgia State UniversityAtlanta, GA, USA; ^2^Laboratoire de Psychologie Cognitive, Aix-Marseille UniversitéMarseille, France; ^3^Division of Developmental and Cognitive Neuroscience, Yerkes National Primate Research CenterAtlanta, GA, USA

**Keywords:** imitation recognition, mirror self recognition, social cognition, chimpanzees, imitation

## Abstract

Imitation recognition provides a viable platform from which advanced social cognitive skills may develop. Despite evidence that non-human primates are capable of imitation recognition, how this ability is related to social cognitive skills is unknown. In this study, we compared imitation recognition performance, as indicated by the production of testing behaviors, with performance on a series of tasks that assess social and physical cognition in 49 chimpanzees. In the initial analyses, we found that males were more responsive than females to being imitated and engaged in significantly greater behavior repetitions and testing sequences. We also found that subjects who consistently recognized being imitated performed better on social but not physical cognitive tasks, as measured by the Primate Cognitive Test Battery. These findings suggest that the neural constructs underlying imitation recognition are likely associated with or among those underlying more general socio-communicative abilities in chimpanzees. Implications regarding how imitation recognition may facilitate other social cognitive processes, such as mirror self-recognition, are discussed.

## INTRODUCTION

An important form of human cognition and learning is imitation. Imitation is defined as reproducing an action after seeing it performed (modified from Thorndike, 1898). It has been suggested by some that imitative learning is a uniquely human form of learning and this claim has stimulated a significant body of research in non-human animals, and particularly non-human primates (Hayes and Hayes, 1952; Tomasello et al., 1987, 1993; Custance et al., 1995; Voelkl and Huber, 2000; Myowa-Yamakoshi et al., 2004; Call et al., 2005; Horner and Whiten, 2005; Tennie et al., 2006; Bard, 2007; Buttelmann et al., 2007; Hobaiter and Byrne, 2010). Further, the neural constructs underlying imitation appear to be rooted in a proposed action-recognition system of mirror neurons that encode both the somatosensory input of another individual performing an action as well as the motor output required for the individual to perform the action themselves. This mirror neuron system (MNS), in essence, allows for the deciphering of the actions, goals, and mental states of a social partner by mapping them onto familiar counterparts that express the same motor repertoires (see Iacoboni, 2009 for review).

Beyond imitation, as an action-understanding mechanism, the MNS has been hypothesized to reflect a ‘like-me’ recognition of other humans as sentient beings (Meltzoff, 2002; Meltzoff and Prinz, 2002). Thus, the MNS has been hypothesized to underlie other social cognitive processes that are predicated on the “like-me” system such as empathy, theory of mind, sympathy, and joint attention (Meltzoff and Decety, 2003; Decety, 2010). In support of this hypothesis are cross-sectional and longitudinal developmental data from children showing significant associations between imitation skills and a variety of other socio-communicative abilities such as empathy (Chartrand and Bargh, 1999), joint attention (Carpenter et al., 1995; Charman et al., 2000), expressive language (Tomasello et al., 1993; Slaughter and McConnell, 2003) and, of special interest, mirror self-recognition (MSR; Asendorpf et al., 1996; Nielsen and Dissanayake, 2004). The adaptive significance of recognizing oneself in a mirror is difficult to imagine. However, if we consider that MSR is dependent on mapping the visual input of one’s own actions onto motor output and imitation is dependent on mapping the visual input of another’s actions onto motor output, their association is logical. Given the functional activation of the MNS during MSR in humans, (Uddin et al., 2005, 2007), it is not implausible that the MNS may have evolved to serve imitative and social cognitive purposes with MSR abilities arising as a byproduct.

The MNS’s involvement in social exchanges is not unidirectional: the same mechanisms that allow us to reproduce another individual’s actions (i.e., imitation production) presumably underlie the ability to recognize when we are being imitated by another individual (herein referred to as imitation recognition; Decety et al., 2002; Nadel, 2002). Imitation recognition is divided into two categories: implicit recognition, whereby the individual being imitated simply directs their gaze toward an imitative experimenter rather than a non-imitative experimenter and explicit recognition, whereby the individual being imitated employs atypical behaviors to assess the actions of the imitator. For example, testing behaviors are defined as unexpected and sudden behaviors performed by the subject while gazing at the imitator (Asendorpf et al., 1996; Meltzoff, 2002; Nielsen et al., 2006). These aptly named behaviors are produced as a means of testing the contingency between the imitator and the imitatee, much like contingency actions often described in children (24 month olds) and apes when looking into a mirror (Gallup, 1970; De Veer and Van Den Bos, 1999; Bard et al., 2006). Implicit imitation recognition has been reported in rhesus (Paukner et al., 2005) and capuchin monkeys (Paukner et al., 2009) while both implicit and explicit imitation recognition has been reported in human children (implicit: 9 month olds; explicit: 14 month olds, 18 month olds) and great apes (Meltzoff, 1990; Asendorpf et al., 1996; Agnetta and Rochat, 2004; Nielsen et al., 2005; Haun and Call, 2008). Explicit testing behaviors employed by humans and apes but not monkeys suggest that advanced imitation recognition capacities may be a derived trait (Gallup, 1977; Bates and Byrne, 2010). Further, a recent cortical connectivity study comparing monkey, chimpanzee, and human MNSs suggests that species differences in imitative abilities may have a neurological foundation based on homologous but differential white matter connections between MNS regions in each species (Hecht et al., 2013).

Despite the heuristic value of the MNS when considered within the context of comparative studies on imitation, the collective findings appear somewhat paradoxical. For instance, mirror neurons were first discovered in macaque monkeys (see Rizzolatti and Luppino, 2001; Rizzolatti and Craighero, 2004), a species for which there is little if any evidence of imitation (but see Ferrari et al., 2006, 2009). With the exception of neonatal imitation in macaques and chimpanzees (Myowa-Yamakoshi et al., 2004; Ferrari et al., 2006; Bard, 2007), evidence of true imitation, such as that demonstrated in “do-as-I-do” types of tasks are rare in the non-human literature (Hayes and Hayes, 1952; Moore, 1992; Custance et al., 1995; Topal et al., 2006; Abramson et al., 2013). Indeed, though a number of studies have demonstrated that non-human primates can learn to solve certain problem-solving tasks by observation, whether these skills are acquired by imitation or other related processes such as emulation and social facilitation remains a topic of considerable debate (Tomasello et al., 1987; Myowa-Yamakoshi and Matsuzawa, 1999; Voelkl and Huber, 2000; Call et al., 2005; Horner and Whiten, 2005; Buttelmann et al., 2007; Carpenter and Call, 2009; Hobaiter and Byrne, 2010). However, the importance of imitation and imitation recognition in primate social interactions becomes apparent when we consider their probable roles in understanding the actions and intentions of others.

The focus of the current study was twofold. First, we sought to replicate and extend previous studies on imitation recognition in chimpanzees by testing a larger sample of individuals as a means of assessing individual differences in performance. Previous studies have tested rather small samples of subjects [between 1 and 11 apes from three different *genera* Nielsen et al. (2005), Haun and Call (2008)] and here we sought to test a larger cohort within a single ape *genus* in order to better assess individual differences and the role that sex might have on performance. The second goal was to examine the potential association between imitation recognition and individual differences in social and non-social cognition. A number of studies in typically and atypically developing children have shown that individual differences in social cognitive processes, like play and joint attention, are associated with performance on imitation tasks (Carpenter et al., 1995, 2002; Asendorpf et al., 1996; Charman et al., 2000; Rogers et al., 2003; Nielsen and Dissanayake, 2004; Ingersoll and Schreibman, 2006; Whalen et al., 2006; Hobson and Hobson, 2007). It has been well documented that chimpanzees, and other great apes, engage in some aspects of joint attention and related socio-communicative skills (Leavens et al., 2008; Leavens and Racine, 2009; Leavens, 2012); however, unlike studies in developing children, to what extent imitation performance might be associated with socio-communicative abilities is unknown. While a recent study indicated that infant rhesus macaques produce more affiliative behaviors when an experimenter is imitating them than when producing repetitive behaviors (Sclafani et al., 2014), the relationship between social and imitative abilities in primates is largely unexplored. Here, we initially tested chimpanzees on an imitation recognition task and characterized each individual as performing well or poorly based on how consistently they produced testing behaviors in response to being imitated. We subsequently compared these groups on their previously collected Primate Cognition Test Battery (PCTB) performance. The PCTB is a series of tasks that has been previously used to assess social and non-social cognition in humans, apes, and monkeys (Herrmann et al., 2007, 2010a,b; Russell et al., 2011; Schmitt et al., 2011; Hopkins et al., 2014b). Among these, the PCTB was used to demonstrate differential factor structures underlying the cognitive processes of chimpanzees and human children (2 years of age; Herrmann et al., 2010b) as well as the heritability of cognition in chimpanzees (Hopkins et al., 2014b). We predicted that if imitation recognition is associated with other socio-communicative abilities in chimpanzees, rather than a distinct process, then subjects that perform well on the imitation recognition tasks will perform significantly better on socially oriented PCTB tasks than apes that performed poorly.

## MATERIALS AND METHODS

### SUBJECTS

Subjects were 16 male and 33 female chimpanzees housed in social groups ranging from 2 to 12 individuals (with the exception of one singly housed male) at the Yerkes National Primate Research Center. Group sizes remained highly consistent between imitation recognition and PCTB test dates, only two subjects’ group sizes changed and these were only by +1 and -2. The subjects ranged in age from 15 to 44 years of age. All of the chimpanzees have been part of a series of behavioral and cognitive studies (Leavens and Hopkins, 1998; Russell et al., 2011; Lyn et al., 2013; Hopkins et al., 2014a; Latzman et al., 2014) but had not been previously tested for imitation recognition prior to this study. All behavioral tests were approved by the local Institutional Animal Care and Use Committee and complied with the Institute of Medicine recommendations for ethical use of chimpanzees in research.

### IMITATION RECOGNITION METHODS

Imitation recognition testing took place between October, 2008 and May, 2010. Subjects voluntarily separated from their social groups to participate in each brief test session. Each subject participated in three separate test sessions or blocks that consisted of four 3-min trials. The study was designed so that within a test block, there were two imitation trials (IMs) and two control trials either in an ABBA or BAAB order. In IM trials, the experimenter imitated the subjects’ actions as accurately as possible. Following the methods used by Nielsen et al. (2005), we employed three different types of control trials: (1) contingent non-matching (CNM), in which the experimenter produced non-similar actions (i.e., different body part and movement) in response to subjects’ actions, (2) non-contingent non-matching (NCNM), in which the experimenter performed a series of preconceived actions at a pre-set pace, and (3) no action (NA), in which the experimenter did not perform any actions at all. Each subject was assigned to one of three preset block orders. The same condition (i.e., CNM, CNM) was used for both control trials within a block. In this way, trial presentation order was cross-balanced across subjects.

### IMITATION RECOGNITION ANALYSIS

Each session was videotaped and all behaviors of interest were coded and analyzed for the presence of testing behaviors using playback on a Sony HDV 1080i Digital HD Videocassette Recorder. Behaviors of interest were defined as any behavior that is directed at an object (i.e., cage banging), is directed at the experimenter (i.e., lip pouting at the experimenter), is an unusual self-directed behavior (i.e., patting head) or is a demonstration of body contingency testing (i.e., running back and forth). Common self-directed behaviors such as scratching, general locomotive behaviors such as shifting positions, and looking behaviors were not included. As indicators of imitation recognition, we were specifically interested in the frequency of three classes of behavioral responses including testing sequences (TS), behavior repetitions (BRs), and testing poses (TPs).

Testing sequences were defined (modified from Asendorpf et al., 1996) as a sequence, lasting for a minimum of 10 s, of at least four successive, different behaviors with no more than 5 s between any two successive behaviors. For example, one subject exhibited a 30 s sequence consisting of: pulling on the mesh five times, pausing for 4 s, hitting the mesh with their hand two times, hitting the mesh with their foot four times, performing a handstand, hitting the mesh with their foot three times, performing a quick 180° turn, and then hitting the mesh with their foot 5 times.

Behavior Repetitions were defined as the subject performing the same type of action at least four times with no more than 3 s between any two repetitions. For example, a subject hit the wall 16 times consecutively. For both TS and BR, the subject had to be looking (directing head and gaze) at the experimenter at the beginning of and continually monitoring the experimenter throughout the sequence.

Testing Poses were defined as the subject looking at the experimenter while holding an atypical body posture (i.e., open mouth, head stand) for more than 3 s. For example, a subject held onto the mesh and open mouth stared at the EXP for 6 s. The number of TS, BR, and TP responses in each experimental condition (IM, CNM, NCNM, and NA) was the dependent measure of interest.

Inter-rater reliability was assessed for 10.9% of trials (*n* = 64). Significant Pearson’s correlations were calculated for TSs (*r* = 0.797, *p* < 0.001), BRs (*r* = 0.886, *p* < 0.001), and TPs (*r* = 0.916, *p* < 0.001).

### PCTB METHODS

The same subjects tested for imitation recognition were also tested using a modified version of the PCTB as described in Russell et al. (2011) and Hopkins et al. (2014b). PCTB testing took place from January 2008 through June 2009. Briefly, subjects were administered 12 tasks designed to broadly assess social and physical cognition. A brief description of each task is presented in **Table 1** (see Russell et al., 2011 for full descriptions). Within the PCTB task, eight tasks assessed physical cognition and included spatial memory, object permanence, rotation, transposition, quantity discrimination, causality-visual, causality-noise, and tool properties. Four tasks assessed social cognition by requiring subjects to respond to or produce social cues included pointing to cups, comprehension of pointing, attention state, and point/gaze following. Experimenters were blind to the results of the imitation recognition task.

**Table 1:** PCTB task descriptions.

**Physical cognition tasks**
Spatial memory (three trials)	Assessed subjects’ ability to remember the locations of two food rewards out of three possible locations. Success was achieved by only looking in the two correct locations.
Object permanence (nine trials)	Assessed subjects’ ability to follow a hidden food as it underwent either a single or double displacement; thus, either one or two of a possible three cups were manipulated. Success was achieved by locating the hidden food without searching in the location that was not manipulated.
Rotation (nine Trials)	Assessed subjects’ ability to relocate a hidden food item from among three options following a rotation of all three options on the horizontal plane.
Transposition (nine trials)	Assessed subjects’ ability to track and select a food reward that is hidden in one of three locations. The baited location is then switched with the unbaited locations in one of three ways.
Relative numbers (13 trials)	Assessed subjects’ ability to differentiate between and chose the larger of two quantities of food.
Causality noise (six trials)	Assessed subjects’ ability to differentiate between a baited metal container and an unbaited metal container based on the sound produced when they were shaken. Success was achieved if they chose the baited container.
Causality visual (six trials)	Assessed subjects’ ability to identify which of two boards and which of two cloths were baited. In each trial type, the food caused a visible difference in the baited board or cloth.
Tool properties (six trials)	Assessed subjects’ ability to pull the a functionally correct, baited piece of paper as opposed to one that was not baited or one that was cut into two pieces.
**Social cognition tasks**
Comprehension (six trials)	Assessed subjects’ ability to use an experimenter’s gaze or gaze combined with manual point to identify which of two objects to touch.
Production (four trials)	Assessed subjects’ ability to communicate to an experimenter which location had been baited by another experimenter.
Attentional state (eight trials)	Assessed subjects’ ability to perform communicative signals in the appropriate modality for the experimenter’s gaze; visual if gaze was directed toward the subject and auditory if it was not.
Gaze following (three trials)	Assessed subjects’ ability to follow the experimenter’s upward gaze.

### DATA ANALYSIS

IM tests were performed within each condition (IM vs. CNM, IM vs. NCNM, IM vs. NA). Therefore, in order to test for differences in TS, BR, and TP across the four conditions, we calculated the average number of TS, BR, and TP for all IM conditions and used this as the sole measure. This was done to minimize the number of tests performed on the data and guard against Type I error. Separate mixed model analyses of variance were performed for the TS, BR, and TP behavioral responses. For each analysis, condition was the repeated measure (IM, CNM, NCM, NA) while sex was the between group factor. Alpha was set to *p* < 0.05 and all *post hoc* tests were performed using either Tukey’s Honestly Significant Difference or pairwise LSD tests. For the PCTB test, the percentage of correct responses was computed based on performance across trials.

## RESULTS

### IMITATION RECOGNITION RESULTS

**FIGURE 1:**
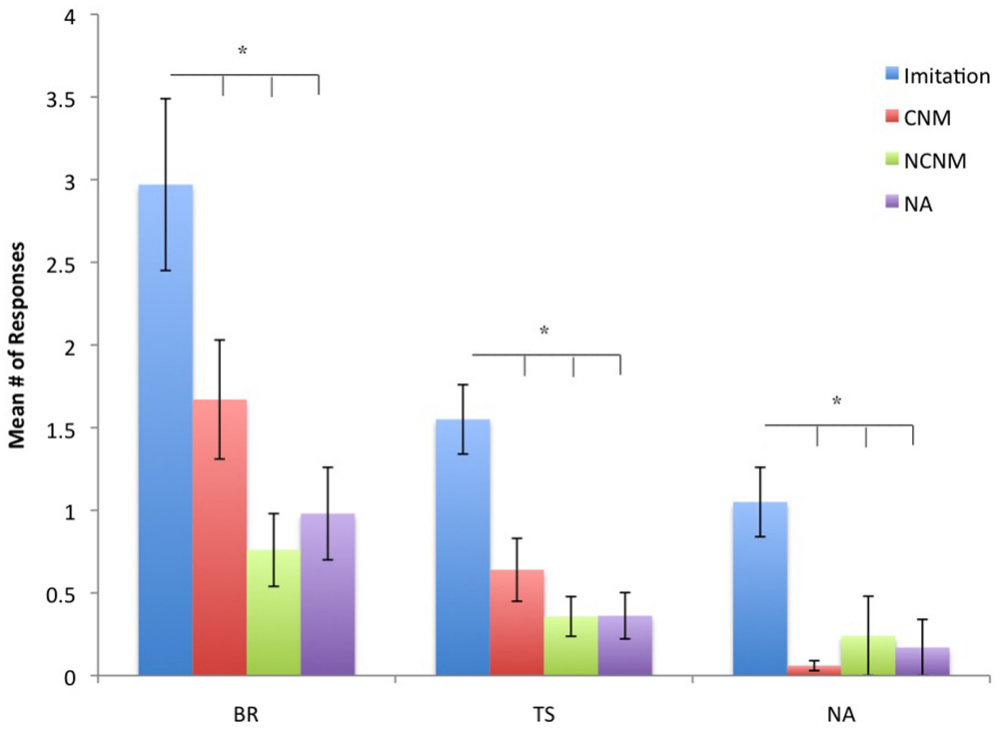
**Mean number of behavior repetitions (BRs), testing sequences (TS), and testing poses (TP) for each condition.** Conditions are imitation, contingent non-matching (CNM), non-contingent non-matching (NCNM), and no action (NA). *p < 0.05.

For TSs *F*(3,141) = 19.70, *p* = 0.001, BRs *F*(3,141) = 11.91, *p* = 0.001, and TPs *F*(3,141) = 13.89, *p* = 0.001, significant differences were found between the experimental and control conditions. The mean number of TSs, BRs, and TPs for each condition are shown in **Figure 1**. For all three types of BRs, *post hoc* analysis indicated that the number of responses in the IM condition were significantly higher than in the CNM, NCNM, and NA conditions (all *p*s < 0.05). We found no significant differences in the frequency in TSs, BRs, and TPs between the CNM, NCNM, and NA conditions. We also found significant sex differences in the frequency of TSs *F*(1,47) = 3.93, *p* = 0.05 (*Mean Male* = 0.975, SE = 0.201 vs. *Mean Female* = 0.482, SE = 0.147) and in the frequency of BRs *F*(1,47) = 4.62, *p* = 0.037 (*Mean Male* = 2.02, SE = 0.415 vs. *Mean Female* = 0.917, SE = 0.302) with males having higher frequencies than females.

### IMITATION RECOGNITION AND PCTB RESULTS

To test for the association between imitation recognition and PCTB performance, we classified the chimpanzees as consistent (CON) or inconsistent (INCON) in their imitation recognition based on the frequency of their TS, BR, and TP responses across the three test conditions. Recall that each chimpanzee received two IM trials within each of the three blocks. Thus, if chimpanzees performed a TS, BR, or TP in two or three of the blocks they were classified as CON. Chimpanzees that produced a TS, BR, or TP in only one or none of tests were classified as INCON. Using this criterion, for TS, there were 24 INCON and 25 CON individuals while there were 22 INCON and 27 CON individuals for the BR behavior. For TP, there were 30 INCON and 19 CON chimpanzees.

**FIGURE 2:**
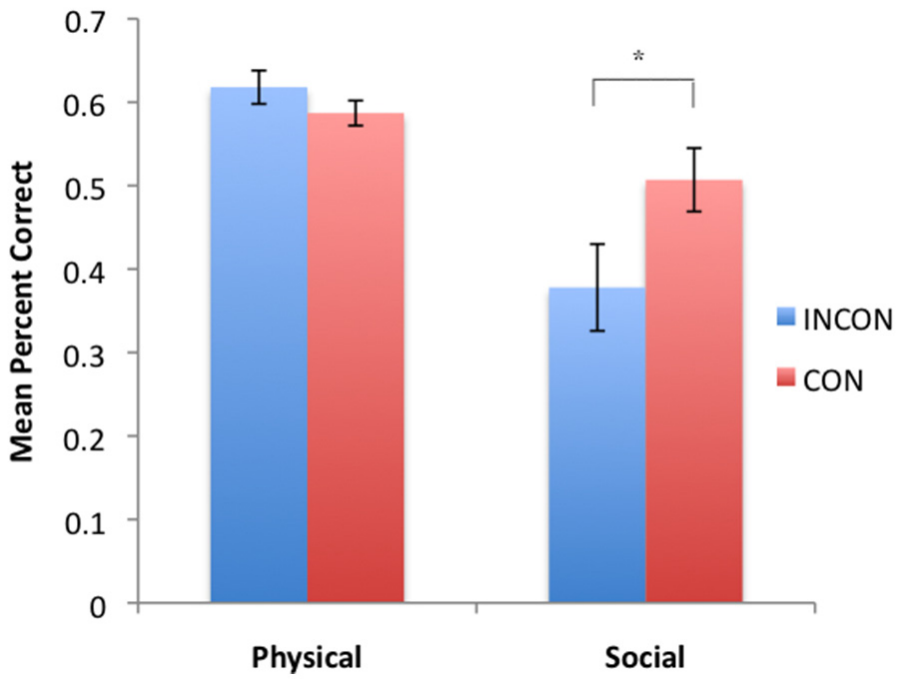
**Mean percent correct in social and physical task groups for subjects classified as either CON or INCON producers of TS in the imitation recognition task.** *p < 0.05.

Based on each subject’s imitation recognition performance for each class of behaviors indicative of imitation recognition, we compared their performance on the PCTB. CON with the original dichotic characterization of PCTB performance (**Table 1**), we computed summary performance scores based on the social and physical cognition tasks. Thus, the performance on the gaze, attention state, production, and comprehension tasks were averaged together to create a global “social” cognition score. Similarly, the performance scores on the spatial cognition, object permanence, rotation, transposition, relative numbers, causality noise, causality visual, and tool properties were averaged together to create a global “physical” cognition score. These social and physical global cognition scores served as the repeated measure while imitation performance classification (CON, INCON) and sex (Male, Female) served as between group factors. For TSs *F*(1,45) = 7.37, *p* = 0.009, a significant two-way interaction was found between imitation recognition classification and PCTB performance. The mean social and physical performance scores in chimpanzees classified as CON or INCON based on their TS production is shown in **Figure 2**. No significant interactions were found between social and physical PCTB scores and BRs or TPs.

**FIGURE 3:**
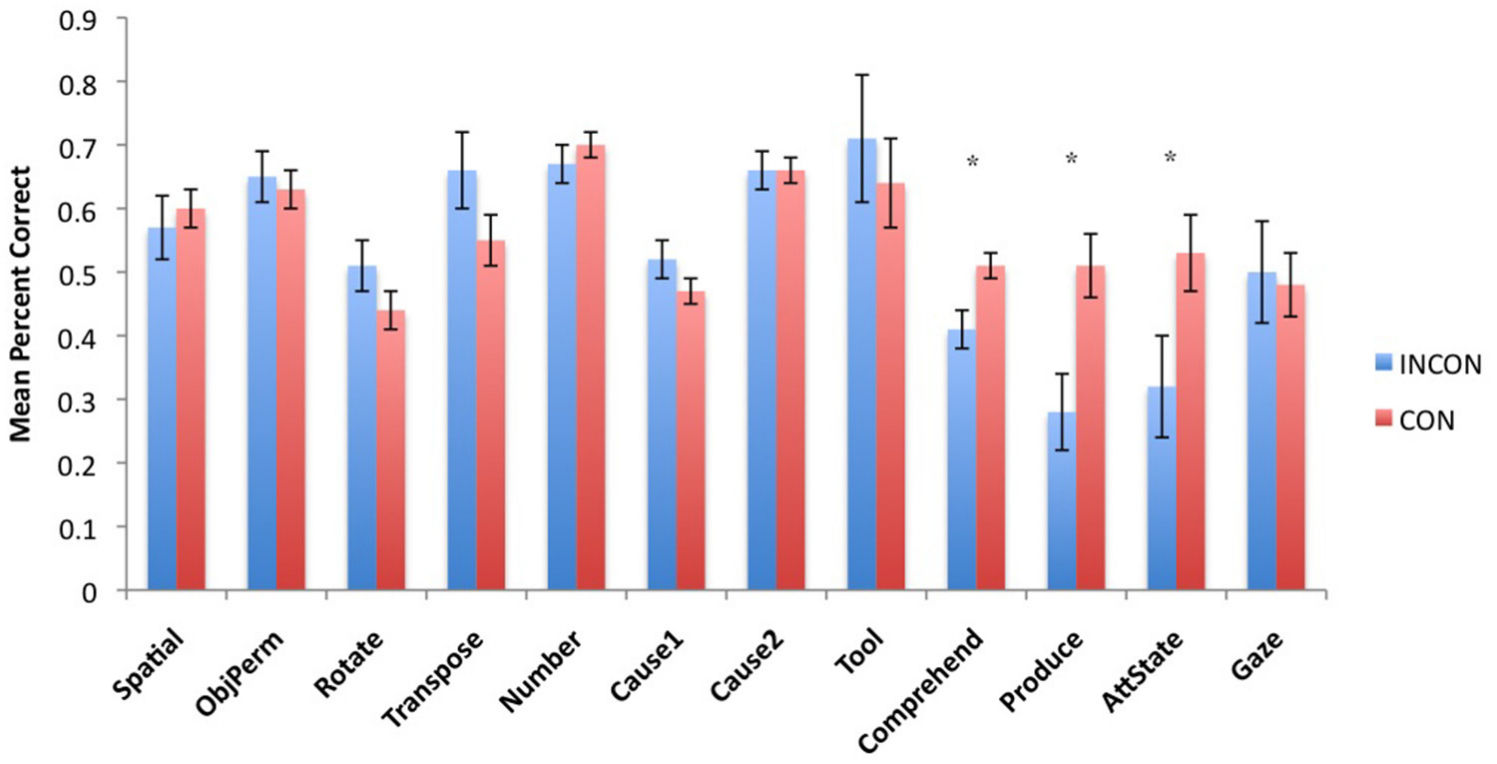
**Mean percent correct in each of the 12 PCTB tasks for subjects classified as either CON or INCON producers of TSs in the imitation recognition task.** *p < 0.05.

**FIGURE 4:**
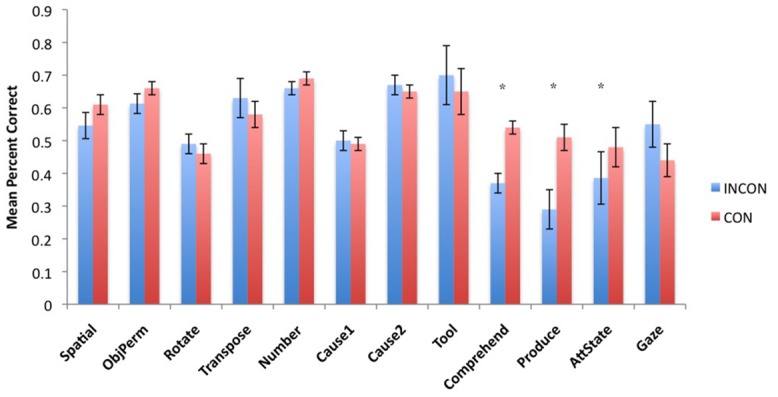
**Mean percent correct in each of the 12 PCTB tasks for subjects classified as either CON or INCON producers of BRs in the imitation recognition task.** *p < 0.05.

Next, to understand the association between imitation recognition measures and individual PCTB task scores, we performed a task-specific analysis with subjects’ performance in each PCTB task serving as the repeated measure while imitation performance classification (CON, INCON) and sex (Male, Female) served as between group factors. For the TS *F*(11,495) = 2.75, *p* = 0.002 and BR *F*(11,495) = 2.31, *p* = 0.009 behaviors, significant two-way interactions were found between imitation recognition classification and PCTB performance. No other significant main effects or interactions were found. *Post hoc* analysis indicated that for chimpanzees classified as CON on the imitation recognition task, as manifest by either TSs and/or BRs, performed better on the gesture production, comprehension, and attention state measures compared to those classified as INCON (all *p*s < 0.05). Performance between the two groups on the remaining tasks was not significantly different from each other. For TPs, there was no significant main effect or interactions with performance on the PCTB tasks. The mean performance score on each PCTB task in chimpanzees classified as CON or INCON based on their TS or BR production are shown in **Figures 3 and 4**, respectively.

## DISCUSSION

### FINDINGS

The present study investigated the relationship between imitation recognition performance and social and physical cognition in chimpanzees and the results are fairly straight-forward. There were two main findings. First, sex differences were evident in imitation recognition with males showing a higher proclivity to respond to being imitated than females. Second, we found that chimpanzees who performed consistently well on two out of three measures of imitation recognition also had significantly higher scores on three out of the four PCTB social cognition tasks than those that performed inconsistently. Performance in non-social cognitive tasks on the PCTB was not associated with imitation recognition performance. These results support our hypothesis that imitation recognition skills are associated with other aspects of socio-communicative abilities in chimpanzees.

Although our results show that imitation recognition and communicative abilities in chimpanzees are related, the nature of this relationship is unclear. Further research should address how practice in social cognitive tasks might influence imitation recognition abilities, or vice versa. We would further note that defining and quantifying imitation recognition was quite challenging. Though we established inter-rater reliability in scoring TSs, BRs, and TPs, this was challenging and does not lend itself to simple interpretations of the chimpanzees’ behavior. Finally, this study used existing PCTB data (Russell et al., 2011; Hopkins et al., 2014b) to measure social and physical cognition but other cognitive measures might have been more sensitive.

### ADAPTIVE SIGNIFICANCE OF IMITATION RECOGNITION

In humans, imitation recognition is suggested to facilitate the interpretation of others’ actions and the understanding of others as cognizant beings via the MNS (Meltzoff, 2002; Meltzoff and Prinz, 2002; Iacoboni, 2009). We suggest that imitation recognition may function similarly, in chimpanzee social cognition, affording imitation recognizers with a potentially better understanding of the actions (and intentions?) of others. However, our results only provide support for the association between imitation recognition and communicative skills in chimpanzees. Whether or not the former facilitates the latter is unknown. Further, while it is tempting to suggest that the behavioral homologies between human and chimpanzee imitation systems might reflect homologies within their respective MNSs, future investigation is necessary.

As has been suggested (Asendorpf and Baudonniere, 1993; Mitchell, 1993, 1997; Nielsen and Dissanayake, 2004), we would concur that imitation recognition may be a critical skill that underlies the well-documented abilities of chimpanzees and other great apes to recognize themselves in mirrors (MSR). When initially confronting a mirror, many chimpanzees engage in contingent actions in front of the mirror (Gallup, 1970; Lin et al., 1992; Povinelli et al., 1997), a behavior that looks quite similar to the TSs or BRs that chimpanzees engage in while being imitated. In truth, the most precise imitator is a mirror. Further, it is of note that only apes evince MSR and show explicit imitation recognition (Haun and Call, 2008; Anderson and Gallup, 2011); thus, heuristically, there is the potential that MSR may be closely related to imitation recognition but this warrants further investigation.

We did not anticipate sex differences and it is unclear why males would be more likely to engage in imitation recognition than females. Nonetheless, in some wild chimpanzee populations, it has been reported that males are more gregarious and have stronger association indices than females (Goodall, 1986; Wrangham, 1986; Mitani et al., 2000). It might be argued that responding to being imitated by engaging in BRs and TSs may reflect this enhanced gregariousness or alternatively an inherent motivation to react to unusual social interactions. Note, the experimenters were female, which conceivably could have lead to increased motivation to participate for male subjects. However, we think this is an unlikely explanation as the same two experimenters conducted the PCTB testing (and many other cognitive and interactive tests Russell et al., 2011; Hopkins and Taglialatela, 2013) in which no sex differences were found. It could also be suggested that male chimpanzees are simply more attentive and reactive to socio-communicative behaviors and this has some advantage in terms of their development and maintenance of social relationships. For instance, Hopkins et al. (2012) and Latzman et al. (2014) have recently found sex dependent influences of a vasopressin receptor gene (AVPR1A) on personality ratings in chimpanzees. Males with a duplication copy of the RS3 coding regions within the AVPR1A were rated as more dominant than those without a copy. It has also been reported that males with the RS3 copy of the AVPR1A gene performed significantly better than males without a copy on a task assessing receptive joint attention, a simple measure of social cognition (Hopkins et al., 2014a). It is intriguing to speculate that males with the RS3 copy might be simply more prosocial and this potentially manifests itself within the context of MSR or related abilities such as imitation recognition. Although our sample was small, when we examined the mean number of TSs, BRs, or TPs in imitation conditions, we found that RS3+ males (*Mean* = 2.52, SE = 0.69) showed a higher number of responses than RS3+ females (*Mean* = 0.802, SE = 0.448), RS3- males (*Mean* = 2.39, SE = 55), and RS3- females (*Mean* = 1.57, SE = 0.42). These differences were not significant but the patterns of results were in the right direction and perhaps with a larger sample, these results might reveal some significant genetic effects on imitation recognition in chimpanzees.

## CONCLUSION

The importance of imitative capacities in the human social environment is apparent. By providing evidence for this same association in non-human primates, the current study sets the stage for elucidating the origins of the advanced imitative and social communication found in humans. We argue that the presence of imitation skills in non-human primates may have been driven by the role of the MNS in understanding others as sentient beings. Furthermore, we highlight the likely correspondence between the evolution of imitation and MSR abilities (Sowden and Shah, 2014). We propose that the discovery of a link between imitation and social skills in chimpanzees could suggest that a common neural substrate for these abilities may have been present in our most common ancestor.

## Conflict of Interest Statement

The authors declare that the research was conducted in the absence of any commercial or financial relationships that could be construed as a potential conflict of interest.
